# Occult Disco-Ligamentous Lesions of the Subaxial c-Spine—A Comparison of Preoperative Imaging Findings and Intraoperative Site Inspection

**DOI:** 10.3390/diagnostics11030447

**Published:** 2021-03-05

**Authors:** Insa Janssen, Nico Sollmann, Melanie Barz, Thomas Baum, Karl Schaller, Claus Zimmer, Yu-Mi Ryang, Jan S. Kirschke, Bernhard Meyer

**Affiliations:** 1 Department of Neurosurgery, Geneva University Hospitals, Rue Gabrielle-Perret-Gentil 4, 1205 Geneva, Switzerland; Karl.Schaller@hcuge.ch; 2 Department of Neurosurgery, School of Medicine, Klinikum rechts der Isar, Technical University of Munich, Ismaninger Str. 22, 81675 Munich, Germany; Melanie.Barz@tum.de (M.B.); Yu-Mi.Ryang@helios-gesundheit.de (Y.-M.R.); Bernhard.Meyer@tum.de (B.M.); 3 Department of Diagnostic and Interventional Neuroradiology, School of Medicine, Klinikum rechts der Isar, Technical University of Munich, Ismaninger Str. 22, 81675 Munich, Germany; Thomas.Baum@tum.de (T.B.); Claus.Zimmer@tum.de (C.Z.); Jan.Kirschke@tum.de (J.S.K.); 4 TUM-Neuroimaging Center, Klinikum rechts der Isar, Technical University of Munich, 81675 Munich, Germany; 5 Department of Diagnostic and Interventional Radiology, University Hospital Ulm, Albert-Einstein-Allee 23, 89081 Ulm, Germany; 6 Department of Neurosurgery, Helios Klinikum Berlin-Buch, Schwanebecker Chaussee 50, 13125 Berlin, Germany

**Keywords:** cervical spine trauma, computed tomography, degenerative cervical spondylosis, disco-ligamentous injuries, intervertebral disc, magnetic resonance imaging

## Abstract

Despite the general acceptance of magnetic resonance imaging (MRI) as the gold standard for diagnostics of traumatic disco-ligamentous injuries in the subaxial cervical spine, clinical experience shows cases where no lesion is detected in MRI exams but obtained during surgery. The aim of this study was to compare intraoperative site inspection to preoperative imaging findings and to identify radiological features of patients having a risk for under- or over-estimating disco-ligamentous lesions. We performed a retrospective analysis of our clinical database, considering all patients who underwent surgical treatment of the cervical spine via an anterior approach after trauma between June 2008 and April 2018. Only patients with availability of immediate preoperative computed tomography (CT), 3-Tesla MRI scans, and information about intraoperative findings were considered. Results of preoperative imaging were set in context to intraoperative findings, and receiver operator characteristics (ROC) were calculated. Out of 144 patients receiving anterior cervical surgery after trauma, 83 patients (mean age: 59.4 ± 20.5 years, age range: 12–94 years, 63.9% males) were included in this study. Included patients underwent surgical treatment via anterior cervical discectomy and fusion (ACDF; 79 patients) or anterior cervical corpectomy and fusion (4 patients) with ventral plating. Comparing preoperative imaging findings to intraoperative site inspection, a discrepancy between imaging and surgical findings was revealed in 14 patients, leading to an overall specificity/sensitivity of preoperative imaging to identify disco-ligamentous lesions of the cervical spine of 100%/77.4%. Yet, adding the existence of prevertebral hematoma and/or vertebral fractures according to preoperative imaging improved the sensitivity to 95.2%. Lack of sensitivity was most likely related to severe cervical spondylosis, rendering correct radiological reporting difficult. Thus, the risk of missing a traumatic disco-ligamentous injury of the cervical spine in imaging seems to be a particular threat in patients with preexisting degenerative cervical spondylosis. In conclusion, incorporating the existence of prevertebral hematoma and/or vertebral fractures can significantly improve diagnostic yield.

## 1. Introduction

Cervical spine injury is common, either isolated or as a concomitant injury in polytrauma, and can lead to devastating morbidity and mortality [1,2]. Typical accident mechanisms associated with bony and soft tissue injuries are high-speed motor vehicle accidents, high-speed sports injuries, or falls [3,4]. The clinical presentation can be heterogeneous, but common symptoms range from upper extremity paresthesia to incomplete or complete tetraplegia.

In the emergency setting of blunt trauma, computed tomography (CT) of the cervical spine is the first-line imaging modality of choice for initial evaluation and potential clearance [5,6]. This is due to the wide disposability and speed of examination of CT, with radiographs being required if CT is yet unavailable [5,6,7]. In case of clinical or radiological concern for further cervical spine injury in terms of traumatic disco-ligamentous injuries including especially anterior longitudinal ligament (ALL), posterior longitudinal ligament (PLL), or intervertebral disc injury, magnetic resonance imaging (MRI) is the gold standard for diagnostic evaluation at the subaxial cervical spine [2,3,8,9,10]. Furthermore, the detection of spinal cord injury, other soft tissue damage, occult osseous lesions, or small hematoma is also possible [11]. Yet, it is assumed that the prevalence of acute cervical spine injury without concomitant bony fractures is low [3,12]. Prompt initial imaging-based diagnostics can be key for correct clinical decision-making, to detect and treat possibly life-threatening complications early, and to plan the surgical procedure [12,13,14,15,16].

Although particularly MRI plays a key role in the evaluation of patients with traumatic injuries of the subaxial cervical spine, clinical experience shows cases where no lesion is described by the radiologist but obtained during surgery. However, to date, there is only a limited body of literature available directly comparing preoperative imaging and intraoperative findings in such patients [3,11,17,18]. An early study investigated 31 patients who underwent preoperative MRI and surgery for acute cervical spine trauma between 1998 and 2001, reporting on >90% sensitivity of MRI for intervertebral disc, PLL, and interspinous soft tissue injury, but stating lower sensitivity for ALL injury (71%) or damage to the ligamentum flavum (67%) [17]. Notably, later studies were inhomogeneous in their study design and evaluation, revealing considerably high variability in these values, with sensitivity ranging between approximately 48% to 100% for ALL and between 50% and 93% for PLL injuries [3,11,17,18]. Yet, such previous work is limited by rather small patient cohorts, has solely considered scanning at lower magnetic fields (1.5 Tesla), and falls short in distinctly exploiting causes for restricted sensitivity with regard to MRI exams [3,11,17,18].

Against this background, the aim of this study was to correlate intraoperative site inspection to preoperative imaging findings, considering both presurgical CT and MRI acquisitions, and to identify radiological features of patients having a risk for under- or over-estimating disco-ligamentous lesions of the subaxial cervical spine.

## 2. Materials and Methods

### 2.1. Study Design and Patient Inclusion

We performed a retrospective analysis of our clinical database at a large university hospital. All patients who underwent surgical treatment of the cervical spine via an anterior approach after trauma between June 2008 and April 2018 were identified. Only patients with (1) availability of both immediate preoperative CT and MRI scans, (2) preoperative MRI performed with a 3-Tesla system using dedicated multi-sequence protocols for suspected cervical trauma, (3) a field of view (FOV) covering at least the entire cervical spine (in preoperative CT and MRI), and (4) availability of detailed information about intraoperative findings (according to surgical reports and intraoperative situs documentation) were included. Patients who did not meet the abovementioned criteria or showed relevant motion artifacts in imaging data (leading to non-diagnostic image quality) were excluded.

We assessed clinical, intraoperative, and radiological findings. The immediate preoperative CT and MRI data were evaluated by three radiologists in consensus reading in the context of the study. Out of 144 patients receiving anterior cervical surgery after trauma in the defined period, 83 patients fulfilled the inclusion criteria and were enrolled in the present study.

### 2.2. Ethical Approval

The ethics committee of the institutional review board (registration number: 238/17 S) approved this study. We obtained patient consent by a general consent form for the storage and analysis of tissue or blood samples, and we anonymized data in the analysis for scientific purposes. The need for written informed consent for this study was waived by the institutional review board due to the retrospective design of the study.

### 2.3. Preoperative Imaging

#### 2.3.1. Computed Tomography

Imaging by CT was performed either in the context of a polytrauma whole-body scan or for dedicated assessment of the whole dorsal or cervical spine only. For each protocol, axial images (slice thickness of 1 mm) as well as coronal and sagittal images (slice thickness of 3 mm), centered on the vertebral column, were reconstructed using a bone kernel. CT was performed with a Siemens scanner (in 72.3% of patients; Somatom Definition AS or AS+ [50 patients], Somatom Sensation 64 or Sensation Cardiac 64 [7 patients], Somatom Sensation 16 or Somatom Emotion 16 [3 patients], Siemens Healthineers, Erlangen, Germany), Philips scanner (in 26.5% of patients; Brilliance 64 [16 patients], iCT 256 [4 patients], Ingenuity Core 128 [1 patient], IQon [1 patient], Philips Healthcare, Best, The Netherlands), or Toshiba scanner (in 1.2% of patients; Activion 16 [1 patient], Toshiba Medical Systems, Otawara, Japan).

#### 2.3.2. Magnetic Resonance Imaging

Imaging by MRI was acquired in supine position using body coils placed over the area of interest and dedicated multi-sequence protocols for trauma patients. Scanning was performed with 3-Tesla Siemens systems (in 24.1% of patients; Magnetom Verio [17 patients], Magnetom Skyra [3 patients], Siemens Healthineers, Erlangen, Germany) or Philips systems (75.9% of patients; Achieva or Achieva dStream [58 patients], Ingenia [5 patients], Philips Healthcare, Best, The Netherlands). Scanning covered only the cervical to upper thoracic spine in 54 patients, the cervico-thoracic spine in 11 patients, and the whole spine in the remaining 18 patients. Table 1 provides an overview of sequences acquired in the study cohort.

### 2.4. Surgery

All patients underwent surgical treatment via an anterior cervical approach. Seventy-nine patients underwent an anterior cervical discectomy and fusion (ACDF), 4 patients received an anterior cervical corpectomy and fusion. Due to the traumatic mechanism a ventral plating was additionally performed in all cases. In a minority of cases, an additional surgery with a second approach for posterior cervical fixation (massa lateralis or pedicle screw fixation) was performed (Table 2).

Indications for surgical treatment were cervical instability due to disco-ligamentous injury proven in cervical MRI with or without vertebral fractures and with or without spinal cord injury in 48 cases. Neurological deficit in terms of a central cord syndrome or traumatic myelopathy in patients with pre-existing cervical spinal stenosis was the indication for surgical treatment in 26 cases. In the remaining cases, bony lesions such as incomplete burst fractures, bilateral fractures of the pedicle, and/or facet joint injury without subluxation required surgical treatment.

### 2.5. Statistical Analyses

For statistical data analysis, GraphPad Prism (version 7.0; GraphPad Software Inc., San Diego, CA, USA) was used. Descriptive statistics were calculated for parameters derived from clinical, intraoperative, and radiological findings, using mean ± standard deviation (SD), ranges, or absolute and relative frequencies.

Receiver operator characteristics (ROC) were calculated using the following definitions based on intraoperative and radiological findings: true positive (TP) = disco-ligamentous injury detected in preoperative imaging AND detected during intraoperative site inspection, true negative (TN) = disco-ligamentous injury absent in preoperative imaging AND absent during intraoperative site inspection, false positive (FP) = disco-ligamentous injury detected in preoperative imaging AND absent during intraoperative site inspection, and false negative (FN) = disco-ligamentous injury absent in preoperative imaging AND detected during intraoperative site inspection. Based on these definitions, we calculated the specificity and sensitivity for preoperative imaging. In addition, a second ROC analysis was performed to analyze the role of prevertebral hematoma and/or vertebral fractures in the context of disco-ligamentous injury, adding the absence or presence of prevertebral hematoma and/or vertebral fractures (on the level of pathology according to intraoperative site inspection) to these definitions.

## 3. Results

### 3.1. Patient Characteristics

**Figure 1:**
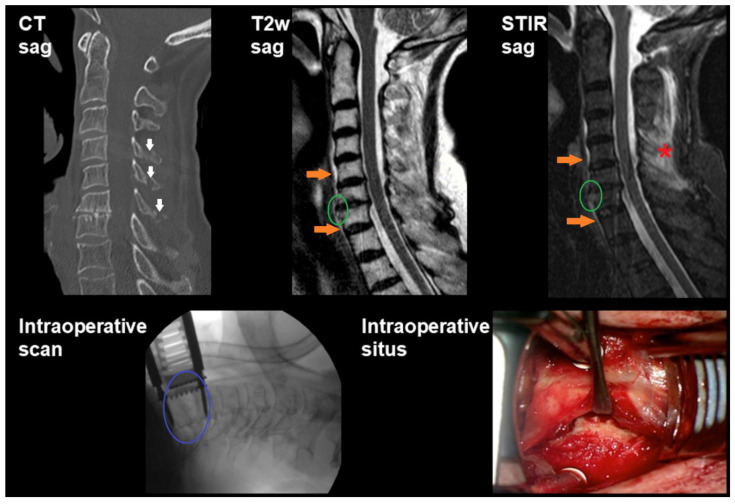
Preoperative imaging shows fractures of the spinous processes C4, C5, and C6 according to computed tomography (CT; *white arrows*). Cervical magnetic resonance imaging (MRI), using sagittal T2-weighted and short tau inversion recovery (STIR) sequences, reveals a small prevertebral hematoma (*orange arrows*) as well as ligamentous injury (*green circles*) and edema in the nuchal soft tissue (*red asterisk* in STIR imaging). Intraoperative X-ray shows a gaped intervertebral space between C6/7 (*blue circle*), indicative of instability at the lower cervical spine. Furthermore, damage to the intervertebral disc between C6/7 as well as a total rupture of the anterior longitudinal ligament (ALL) were revealed during intraoperative situs inspection according to microscopic view during surgery (depicted is the surgical access route and traumatically damaged tissue in the context of surgical stabilization due to disco-ligamentous injury). Indication for surgical treatment was based on neurological deficits in terms of a central cord syndrome.

**Figure 2:**
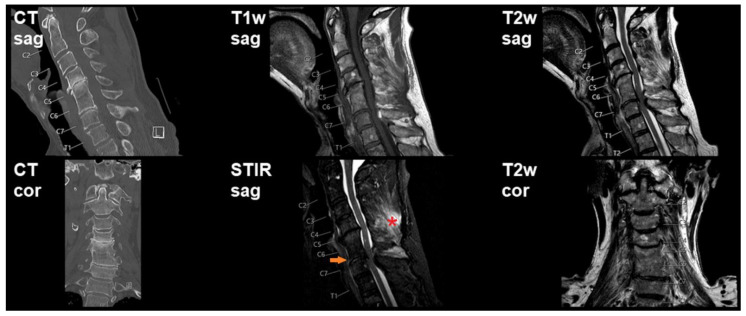
Preoperative imaging shows degenerative changes particularly for the segments C4/C5 and C5/C6 according to computed tomography (CT). Cervical magnetic resonance imaging (MRI), using T1-weighted, T2-weighted, and short tau inversion recovery (STIR) sequences, depicts a small prevertebral hematoma (*orange arrow* in STIR imaging) as well as ligamentous injury and edema in the nuchal soft tissue (*red asterisk* in STIR imaging). Furthermore, spinal stenosis is present at level C6/C7.

**Figure 3:**
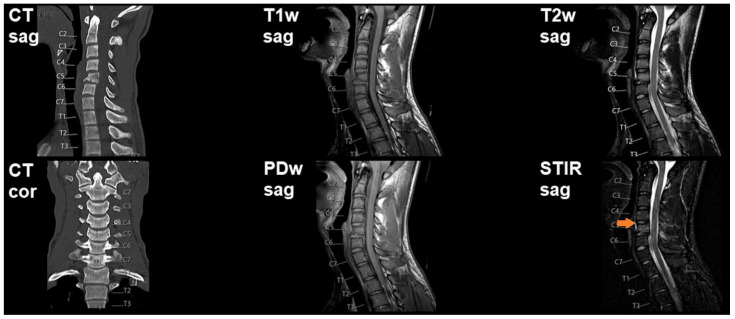
Preoperative imaging shows a fracture of the vertebral body of C5 according to computed tomography (CT). Cervical magnetic resonance imaging (MRI), using T1-weighted, T2-weighted, proton-density (PD)-weighted, and short tau inversion recovery (STIR) sequences, is suggestive of a subtle prevertebral hematoma (*orange arrow* in STIR imaging), but does not clearly reveal a ligamentous injury.

Overall, 83 patients (mean age: 59.4 ± 20.5 years, age range: 12–94 years, 63.9% males) were retrospectively included in this study. Table 2 provides an overview of demographics and clinical details, Figure 1, Figure 2 and Figure 3 show illustrative patient cases.

### 3.2. Imaging and Intraoperative Findings

In 48 patients (57.8%), a disco-ligamentous injury was shown or suspected in preoperative imaging and confirmed intraoperatively. During surgery, a disco-ligamentous injury was intraoperatively observed in 62 patients (74.7%) in total.

Comparing preoperative imaging findings to intraoperative site inspection, we observed 48 TP, 21 TN, 0 FP, and 14 FN incidences, meaning that there was a discrepancy between imaging and surgical findings in 14 patients. Yet, when incorporating prevertebral hematoma and/or vertebral fractures on the level of surgically observed disco-ligamentous injury, the FN fraction decreased to 10 and 3 cases, respectively. Most affected segments of FN incidences were C6/7 (8 patients) and C5/6 (6 patients). In the majority of cases an injury was obtained intraoperatively for a single level (9 patients), yet in five cases two levels were affected. Table 3 provides details for the 14 patient cases assigned to the FN fraction.

### 3.3. Sensitivity and Specificity

The overall specificity/sensitivity of preoperative imaging to identify disco-ligamentous lesions of the cervical spine was 100%/77.4%, indicating a relevant risk to miss a disco-ligamentous injury. When also considering prevertebral hematoma as detected on preoperative imaging (on the level of confirmed disco-ligamentous injury according to intraoperative site inspection), the sensitivity increased to 83.9%. Considering both the existence of prevertebral hematoma and vertebral fractures (on the level of confirmed disco-ligamentous injury according to intraoperative site inspection), sensitivity further increased to 95.2%. Thus, considering both a prevertebral hematoma and vertebral fractures may considerably increase the sensitivity of MRI in detecting disco-ligamentous injuries.

## 4. Discussion

This study’s aim was to compare preoperative imaging findings derived from CT and MRI to intraoperative site inspection in patients with a clinical suspicion of disco-ligamentous injury, who underwent surgical treatment of the cervical spine via an anterior approach after trauma. While specificity was 100%, sensitivity for detecting disco-ligamentous injury by preoperative imaging was 77.4% among the 83 patients enrolled. Yet, adding prevertebral hematoma and/or vertebral fractures considerably increased the sensitivity to detect disco-ligamentous injury to 83.9% and 95.2%, respectively.

For the cervical spine, previous research has shown considerably high variability in values for sensitivity, reporting ranges between approximately 48% to 100% for ALL, 50% to 93% for PLL, and about >90% for intervertebral disc injuries, respectively [3,11,17,18]. In this context, a study by Malham et al. comparing cervical MRI and intraoperative findings in 31 patients showed high FN rates for both ALL and PLL injury, with sensitivity as low as 48% and 50%, respectively [11]. In a case–control design with 21 acute spinal trauma patients, MRI was 100% sensitive in detecting injury to the intervertebral discs, ALL, and interspinous ligaments, and moderately sensitive for PLL injury (80%) [18]. Recently, a study in 21 patients with suspicion of disco-ligamentous cervical injury after hyperextension trauma demonstrated overall percent agreement between short tau inversion recovery (STIR) or conventional T2-weighted imaging and intraoperative findings of 90.9% and 69.7%, respectively, with an agreement of 87.9% for the ALL and of 78.8% for intervertebral discs, for which STIR imaging always showed higher agreement [3].

In the present study, mostly intervertebral disc injury and damage to the ALL were not detected on preoperative imaging, which turned out to be present during intraoperative site inspection. Regarding the ALL, conventional T2-weighted images are probably not ideal for detecting damage since the distinction between the ligament and adjacent annulus of the intervertebral discs may not be sufficiently possible, thus rendering conventional T2-weighted sequences difficult for evaluation of integrity [17,19]. A similar situation may be of relevance at least for anterior injuries of intervertebral discs with close contact to the ALL, probably making a combined minor injury of both adjacent structures difficult to detect. However, a recent study indicated that STIR sequences may outperform conventional T2-weighted imaging for detection of different disco-ligamentous lesions at the cervical spine [3]. In the present cohort, indeed all patients of the FN fraction underwent imaging by conventional T2-weighted as well as STIR sequences. Regarding the causes of missed pathology, we observed severe degenerative changes in 50% of patients of this fraction, which have most likely contributed to hampered imaging-based detection of injury. Furthermore, for the other 50%, causes for a lack of sensitivity may be related to a lack in spatial resolution for MRI and/or only subtle injury-related changes not visible during assessment of imaging. However, it should be noted that in 11 out of the 14 patients of the FN proportion, other trauma-related imaging findings were indeed detected (e.g., vertebral fracture, hematoma) or at least one site of ligamentous injury was suggested (e.g., damage to interspinous ligaments only), possibly increasing awareness of potential additional trauma to the ALL and/or intervertebral discs that are however occult in presurgical imaging.

Since missing a disco-ligamentous injury of the cervical spine during imaging is potentially associated with devastating clinical consequences ranging from neurological deficits to cervical deformation and chronic pain in the long term, the limited sensitivity of preoperative imaging to identify disco-ligamentous lesions of the cervical spine should be taken into account for treatment decision-making. Regarding the possible factors influencing the unequivocal identification of all disco-ligamentous injuries compared to intraoperative findings in our patient cohort, we observed that the presence of severe degenerative changes leads to restrictions for diagnostic yield of preoperative imaging. Thus, in patients with severe degenerative cervical spondylosis, adequate trauma mechanism, or moderate bony and/or soft tissue injuries on CT and MRI, disco-ligamentous injuries should be taken into consideration in correlation with clinical presentation, even if not directly visible on MRI. In case of a suspicion of injury to the ALL, PLL, and/or intervertebral discs, first of all the distinct indication should be specified and communicated with the radiologist in order to choose an adequate imaging protocol to reduce the probability of errors due to suboptimal image acquisition. Yet, our findings show that presence of a prevertebral hematoma and/or vertebral fractures on the level of disco-ligamentous injury (according to intraoperative findings) are highly suggestive for a disco-ligamentous injury in cases where ligamentous discontinuity or evident disc trauma is not explicitly visible in MRI, suggesting that rigorous interpretation of instability only when such pathology is captured by MRI should be made with high caution. Specifically, including the presence of prevertebral hematoma and/or vertebral fractures on the level of surgically confirmed disco-ligamentous injury increased the sensitivity to values of 83.9% and 95.2%, respectively. Given the highly preselected cohort of the present study (the majority of patients have been operated on due to the evidence of potentially instable injuries), these values exceed those of previous publications.

While different sequence protocols were acquired for detection of suspected disco-ligamentous injury in the present study, all imaging was derived from 3-Tesla systems, which is in contrast to scanning at lower field strengths as investigated in previous work (1.5 Tesla) [3,11,17,18]. Yet, distinct sequence protocols were variable, being a result of investigations on different MRI systems and over a longer period in time considered for enrollment. In this context, there seems no doubt about the requirement for MRI of the cervical spine for diagnostic evaluation of traumatic injuries in the subaxial cervical spine as the gold-standard method [2,3,8,9]. However, there is no definite consensus on the exact MRI protocol to be used in patients suffering from such injury. A multi-sequence approach consisting of at least sagittal T1- and T2-weighted spin echo sequences and STIR sequences, supplemented by axial T2-weighted sequences tailored to the levels of pathology, seems routine [9,20,21]. Particularly STIR sequences have already been recommended as highly relevant for the evaluation of disco-ligamentous injuries, and particularly for damage to the ALL at the cervical spine [3,9]. A more advanced imaging approach that could become highly relevant to MRI in suspected spinal trauma may be the DIXON technique in combination with turbo spin echo. Specifically, it may be an alternative to consecutive acquisitions of sagittal T1- and T2-weighted and STIR sequences in a row because, as the DIXON technique makes advantage of chemical shift for water and fat separation, it can deliver separate image datasets within a single sequence [21,22,23]. Specifically, a T2-weighted DIXON sequence provides four image sets that differ in their contrasts, comprising so-called fat-only images (comparable to standard T1-weighted sequences), water-only images (comparable to STIR sequences), in-phase images (comparable to standard T2-weighted sequences), and out-of-phase images [21,22,24,25]. Acquiring T2-weighted DIXON sequences may allow to skip dedicated T1-weighted, T2-weighted, and STIR sequences, saving scan time and limiting the duration of time inside the scanner for trauma patients, which could limit motion artifacts related to long acquisition times. Furthermore, simultaneous to such time savings, the DIXON method could generate images with an elevated signal-to-noise ratio compared to STIR imaging and more homogeneous fat suppression [26,27,28,29,30,31,32,33]. These advancements may increase the diagnostic yield of preoperative MRI in suspected disco-ligamentous injury; however, due to the novelty of the approach on commercially available and clinically used MRI systems, data for comparison to standard protocols is currently mostly lacking in trauma patients. This is also resembled in our cohort with only one patient having DIXON imaging available, which is related to the time of enrollment of our patients.

Flexion-extension radiography of the cervical spine was carried out only sporadically in our cohort and did not provide any relevant additional information for treatment decision-making, thus was not considered an integral part of this study’s setup and design. While dynamic cervical radiography is described to have a role in detecting instable fractures of the cervical spine and particularly in unconscious patients after blunt trauma during the acute emergency setting, the reliability for this purpose remains controversial [34]. Recent studies obtained low diagnostic relevance, mainly limited by an insufficient illustration of the lower cervical spine and cervico-thoracic junction at motion less than 30° range [35]. Assuming that the majority of FN incidences in our cohort was located in the segments C5/6 and C6/7, a dynamic radiography seems not reliable as an additional diagnostic tool to MRI.

When interpreting the results of this study, some relevant limitations have to be acknowledged. First, although all MRI data considered were uniquely taken from 3-Tesla systems, differences in scanning protocols are evident. Yet, in the context of a retrospective study design, this seems justified and resembles daily clinical practice. Second, the incidences of injuries to interspinous ligaments were very low in our cohort, making our results on ROC rather specific to other injuries including primarily the ALL and intervertebral discs, but also the PLL to a lesser extent. Third, the retrospective character of the study carries the risk of inaccuracies and incompleteness in the documentation of perioperative observations. Furthermore, the investigated patients reflect a preselected cohort as the majority was operated on for the clinical indication of assumed traumatic instability.

## 5. Conclusions

The risk of non-detection of traumatic disco-ligamentous injuries of the cervical spine in MRI seems to be in particular present in patients with preexisting degenerative cervical spondylosis. Yet, when including the existence of prevertebral hematoma and/or vertebral fractures, the diagnostic yield of MRI of the cervical spine can be increased to a specificity of 100% and sensitivity of 95.2%. The review of T2-weighted DIXON sequences for suitability to detect traumatic disco-ligamentous injuries should be considered in the future to decrease scanning time in trauma patients.

## Figures and Tables

**Table 1:** Acquired sequences during preoperative magnetic resonance imaging (MRI).

Sequence		Number of Patients	
Non-contrast T1-weighted	Sagittal	63	53
	Axial		3
	Sagittal & axial		7
T2-weighted	Sagittal	83	3
	Axial		10
	Sagittal & axial		56
	Axial & coronal		2
	Sagittal & axial & coronal		12
STIR	Sagittal	73	63
	Sagittal & axial		2
	Sagittal & coronal		7
	Sagittal & axial & coronal		1
PD-weighted	Sagittal	29	3
	Coronal		17
	Sagittal & coronal		9
Contrast-enhanced T1-weighted	Sagittal	3	2
	Sagittal & axial		1
TIRM	Sagittal	2	
DIXON	Sagittal & coronal	1	

This table provides details on the number of patients in whom specific sequences were acquired during preoperative MRI at 3 Tesla. Imaging protocols include non-contrast and contrast-enhanced T1-weighted, T2-weighted, short tau inversion recovery (STIR), proton-density (PD)-weighted, turbo inversion recovery magnitude (TIRM), and DIXON sequences.

**Table 2:** Patient cohort.

Item		Value
Number of patients		83
Age (mean ± SD & range; in years)		59.4 ± 20.5 (12–94)
Sex (% males/females)		63.9/36.1
Trauma mechanism/entity (% of patients)	Traffic accident	16.9
	Polytrauma	2.4
	Fall	48.2
	Fall (great height)	32.5
Symptoms (% of patients)	None	0.0
	Nuchalgia	41.0
	Brachialgia	0.0
	Myelopathy	3.6
	Monoparesis	6.0
	Isolated sensory disturbance	4.8
	Central cord syndrome	20.5
	Tetraparesis	8.4
	Nuchalgia & brachialgia	2.4
	Nuchalgia & monoparesis	1.2
	Nuchalgia & sensory disturbance	1.2
	Brachialgia & monoparesis	1.2
	Nuchalgia & brachialgia & myelopathy	1.2
	Myelopathy & monoparesis & sensory disturbance	1.2
	Intubated/not assessible	7.3
Surgical procedure (% of patients)	Anterior cervical discectomy and fusion (ACDF) + ventral plate	75.9
	Anterior cervical discectomy or corpectomy and fusion + ventral plate & dorsal stabilization	19.3
	Anterior cervical corpectomy and fusion + ventral plate	4.8

This table provides information on demographic, clinical, and surgical details of the 83 patients enrolled in this study. All included patients underwent surgical treatment of the cervical spine via an anterior approach after trauma.

**Table 3:** Discrepancies between preoperative imaging and intraoperative findings.

#	Affected Segment(s)	Preoperative Imaging							Intraoperative Situs Inspection			
		Disco-Ligamentous Injury				Other Trauma-Related Findings Affecting the Cervical Spine	Other Findings at the Cervical Spine	Acquired Sequences	Disco-Ligamentous Injury			
		ALL	Intervertebral Disc	PLL	Interspinous Ligaments				ALL	Intervertebral Disc	PLL	Interspinous Ligaments
**1**	C4/5	-	-	-	-	-	Scoliosis, severe multi-segmental spondylosis, myelomalacia, spinal stenosis	T1w sag/ax, T2w sag/ax, STIR sag	X	X	-	-
**2**	C3/4	-	-	-	-	Prevertebral hematoma	Forestier’s disease, severe multi-segmental spondylosis, myelomalacia, spinal stenosis	T2w sag/ax, STIR sag	X	X	-	-
**3**	C5/6	-	-	-	X	Vertebral fracture	-	T1w ax, T2w sag/ax, STIR sag/cor	-	X	-	-
**4**	C5/6	-	-	-	X	Prevertebral hematoma	-	T1w sag, T2w ax, STIR sag/ax/cor, T1w sagittal (CE)	X	X	-	-
**5**	C6/7	-	-	-	-	Vertebral fracture	-	T1w sag, T2w sag/ax, STIR sag, PDW cor	X	X	-	-
**6**	C5/6, C6/7	-	-	-	-	Vertebral fracture	Severe multi-segmental spondylosis	T1w sag, T2w ax/cor, STIR sag	X	X	-	-
**7**	C5/6, C6/7	-	-	-	X	Prevertebral hematoma, vertebral fracture	Severe multi-segmental spondylosis, myelomalacia, spinal stenosis	T1w sag, T2w sag/ax/cor, STIR sag	-	X	-	X
**8**	C2/3	-	-	-	-	Vertebral fracture	Spinal stenosis	T2w ax, STIR sag	X	X	-	-
**9**	C6/7	-	-	-	X	-	Scoliosis, severe multi-segmental spondylosis, myelomalacia, spinal stenosis	T1w sag, T2w sag/ax/cor, STIR sag	X	X	-	-
**10**	C4/5, C5/6	-	-	-	-	Prevertebral hematoma	Forestier’s disease, severe multi-segmental spondylosis, myelomalacia, spinal stenosis	T1w sag, T2w sag/ax, STIR sag	-	X	-	-
**11**	C6/7	-	-	-	-	Vertebral fracture	-	T1w sag, T2w sag/ax, STIR sag	X	X	-	-
**12**	C6/7, C7/T1	-	-	-	-	Vertebral fracture	-	T1w sag, T2w sag/ax, STIR sag	X	X	-	-
**13**	C6/7	-	-	-	X	-	Myelomalacia, spinal stenosis	T1w ax, T2w sag/ax, STIR sag	-	X	-	-
**14**	C5/6, C6/7	-	-	-	X	Vertebral fracture	Scoliosis, severe multi-segmental spondylosis	T1w sag, T2w sag/ax, STIR sag	X	X	-	-

This table provides information on the affected segment(s) of trauma according to preoperative imaging and the injured structures including the anterior longitudinal ligament (ALL), posterior longitudinal ligament (PLL), intervertebral discs, and inter-spinous ligaments. Furthermore, presence of vertebral fractures and/or prevertebral hematoma is given, together with concomitant imaging findings at the cervical spine. The sequences acquired during preoperative magnetic resonance imaging (MRI) at 3 Tesla included non-contrast and contrast-enhanced (CE) T1-weighted, T2-weighted, and/or short tau inversion recovery (STIR) sequences in the axial (ax) and/or sagittal (sag) and/or coronal (cor) plane for the 14 patients with discrepant findings between preoperative imaging and intraoperative site inspection.

## Data Availability

The raw data supporting the conclusions of this article are available on reasonable request from the authors.

## Abbreviations

ACDF	Anterior cervical discectomy and fusion
ALL	Anterior longitudinal ligament
CT	Computed tomography
FN	False negative
FOV	Field of view
FP	False positive
MRI	Magnetic resonance imaging
PD	Proton density
PLL	Posterior longitudinal ligament
ROC	Receiver operator characteristics
SD	Standard deviation
STIR	Short tau inversion recovery
TIRM	Turbo inversion recovery magnitude
TN	True negative
TP	True positive
